# Association of Alanine Aminotransferase and Periodontitis: A Cross-Sectional Analysis—NHANES 2009–2012

**DOI:** 10.1155/2016/3901402

**Published:** 2016-02-11

**Authors:** R. Constance Wiener, Usha Sambamoorthi, Richard J. Jurevic

**Affiliations:** ^1^Dental Practice and Rural Health, School of Dentistry, West Virginia University, Robert C. Byrd Health Sciences Center, Addition 104A, P.O. Box 9448, Morgantown, WV 26506, USA; ^2^Department of Pharmaceutical Systems and Policy, West Virginia University School of Pharmacy, Robert C. Byrd Health Sciences Center (North), P.O. Box 9510, Morgantown, WV 26506-9510, USA; ^3^School of Dentistry, West Virginia University, Health Sciences Center, Addition 102A, P.O. Box 9448, Morgantown, WV 26506, USA

## Abstract

*Objective*. Alanine Aminotransferase is an enzyme associated with not only liver diseases, liver conditions, and metabolic syndrome, but also inflammation. Periodontitis is associated with increased cytokines and other markers of inflammation. The purpose of this study is to determine if an independent association between Alanine Aminotransferase and periodontitis exists.* Methods*. Data from the 2009-2010 and 2011-2012 National Health and Nutrition Surveys (NHANES) were combined. Data concerning periodontitis and Alanine Aminotransferase were extracted and analyzed with Rao Scott Chi-square and logistic regressions. Serum Alanine Aminotransferase was dichotomized at 40 units/liter, and periodontitis was dichotomized to the presence or absence of periodontitis.* Results*. In bivariate Chi-square analyses, periodontitis and Alanine Aminotransferase were associated (*p* = 0.0360) and remained significant in unadjusted logistic regression (OR = 1.30 [95% CI: 1.02, 1.65]). However, when other known risk factors of periodontitis were included in the analyses, the relationship attenuated and failed to reach significance (adjusted OR = 1.17 [95% CI: 0.85, 1.60]).* Conclusion*. Our study adds to the literature a positive but attenuated association of serum Alanine Aminotransferase with periodontitis which failed to reach significance when other known, strong risk factors of periodontitis were included in the analysis.

## 1. Introduction

Alanine Aminotransferase is an enzyme that is a catalyst in the transfer of –NH_2_ group from L-alanine to alpha-ketoglutarate creating pyruvate and L-glutamate which are important reactions in the tricarboxylic acid cycle (also known as the Krebs cycle or the citric acid cycle of energy production) [1]. Alanine Aminotransferase is primarily found in hepatocyte intracellular fluid [1]. Serum levels are used to detect hepatic distress and diseases such as chronic viral infections, autoimmune hepatitis, and steatohepatitis, as well as other conditions such as celiac disease, muscle injury, hemochromatosis, and nonalcoholic fatty liver disease [1]. Serum levels also increase with extreme physical exertion, excessive alcohol intake, some medications, and metabolic syndrome [1], the clustering and synergistic effects of interrelated risk factors leading to diseases of chronic inflammation (cardiovascular disease, stroke, and/or diabetes) [2–5]. Many mechanisms and pathways may be involved in elevated serum Alanine Aminotransferase levels; however, chronic inflammation is one potential mechanism for its increase.

Periodontitis is the destruction of the soft and hard oral tissue supporting the teeth as the result of a biofilm (bacteria, fungi, and viruses), genetic susceptibility, and plaque-induced chronic inflammation, among other risk factors [6–13]. Systemic chronic inflammation negatively impacts periodontal health. Periodontitis is a public health concern as it is a common disease affecting 46% of US adults (64.7 million individuals) [14]. Periodontitis affects oral health quality of life, the number of remaining teeth in older adults, and glycemic control in individuals with diabetes and has social and financial impacts.

The purpose of this study is to determine if there exists an independent association of Alanine Aminotransferase and periodontitis. The theoretical basis is that Alanine Aminotransferase levels are increased with inflammation and periodontitis is a disease of chronic inflammation. Researchers using animal models indicated that such an association exists [15–17]. Researchers also determined that an association of periodontitis and Alanine Aminotransferase existed using a convenience sample of 2225 young Japanese male university students [18]. However, the number of participants with periodontitis was small (4.7%) in that study. The rationale for this study is that there is a need for a large, nationally representative study to determine if an association exits between periodontitis and Alanine Aminotransferase. Such knowledge could help inform decisions by medical personnel concerning dental referrals for patients with high Alanine Aminotransferase levels.

The null hypothesis for this research is that the adjusted odds ratio for the association of high levels of serum Alanine Aminotransferase (≥40 units/liter) on periodontitis is the same as the adjusted odds ratio for normal levels of serum Alanine Aminotransferase (<40 units/liter) on periodontitis.

## 2. Materials and Methods

This study was acknowledged by the West Virginia University Institutional Review Board (protocol 14010466515). The study was conducted using the Strengthening the Reporting of Observational studies in Epidemiology (STROBE) guidelines.

### 2.1. Data Source

Data used in this study were from the combined 2009-2010 and 2011-2012 National Health and Nutrition Examination Surveys (NHANES) which were collected by researchers from the Centers for Disease Control and Prevention (CDC). The NHANES surveys had a stratified, multistage probability sampling strategy. Noninstitutionalized civilian individuals were selected for the survey. They provided written informed consent. Each participant was interviewed and had a physical evaluation in NHANES MEC (Mobile Examination Center).

The periodontal examinations were completed by licensed dentists. He or she had an available halogen light, a HuFriedy*™* periodontal probe, and a mouth mirror for each examination. The examination was limited to individuals who were not edentulous and who did not have heart transplants, artificial valves, congenital heart disease, rheumatic fever, hemophilia, kidney disease with dialysis, or a pacemaker.

Alanine Aminotransferase level in serum or plasma was determined by NHANES researchers using a kinetic rate method. Alanine Aminotransferase is a catalyst in the reversible reaction of L-alanine and a-ketoglutarate to pyruvate and L-glutamate. In the analysis, the pyruvate is further reduced. The rate of change in absorbance at 340 nm over time is proportional to the Alanine Aminotransferase level [19]. The details of the NHANES procedures are available at http://www.cdc.gov/nchs/nhanes.htm.

### 2.2. Eligible Study Population

The researchers for this study used a subset of the available data from the NHANES 2009–2012 surveys. Eligible participants were aged 30–69 years and had complete data on periodontitis and serum Alanine Aminotransferase. The eligible sample included 5,758 participants.

### 2.3. Dependent Variable

The periodontal variable was the dependent variable for this study. Periodontitis was dichotomized as a yes/no variable to establish an adequate sample size. Even though several years of NHANES data were combined, there were too few participants in the categories of “mild periodontitis” and “severe periodontitis” who also had Alanine Aminotransferase data to use those categories as separate categories. Instead, the categories were no periodontitis (the reference group) and periodontitis (mild, moderate, or severe) using the definition of periodontitis for population-based surveillance supported by the CDC in partnership with the American Academy of Periodontology [20]. Their more detailed definition of periodontitis includes* “mild periodontitis”* in which there are at least 2 interproximal areas in which the attachment loss is at least 3 mm and there are at least 2 interproximal areas on different teeth in which the probing depth is at least 4 mm or at least one area with a probing depth of 5 mm;* “moderate periodontitis”* in which there are at least 2 interproximal areas on different teeth in which the attachment loss is at least 4 mm or there are at least 2 interproximal areas on different teeth in which the probing depth is at least 5 mm; and* “severe periodontitis”* in which there are at least 2 interproximal areas which are on different teeth in which the attachment loss is at least 6 mm and there is at least 1 interproximal area in which the probing depth is at least 5 mm [20].

### 2.4. Independent Variable and Other Variables

The key-independent variable was serum Alanine Aminotransferase. It was dichotomized at <40 units/liter (the reference group) and ≥40 units/liter. The cut-point at ≥40 units/liter is routinely used and has been previously reported in peer-reviewed literature to determine normal and high levels of Alanine Aminotransferase in adults [21–23].

The other variables that were included in this study to account for confounding or to further explain the variance for periodontitis included sociodemographic and behavioral variables: sex, race/ethnicity, education, age, income to poverty ratio, smoking, body mass index, insurance coverage, presence of diabetes mellitus, and alcohol consumption. The categories for the additional variables were sex (male versus female), race/ethnicity, (non-Hispanic black, Mexican American, and other versus non-Hispanic white), education (high school, less than high school, and some college versus college degree and above), age (45–54, 55 years and above versus 30–44 years), family income to poverty ratio (less than 2 versus 2 and above), smoking status (current, former smoker versus never smoker), alcohol consumption (moderate, heavy versus no consumption), body mass index (25–29, 30 and above versus 0–24), insurance coverage (no versus yes), and diabetes (yes versus no).

### 2.5. Statistical Analyses

Due to the complex study design, the analysis accounted for clustering, stratification, selected sample population, and sample weights. Descriptions of the sample population, bivariate (Rao Scott Chi-square) analyses with periodontitis, and logistic regression analyses were completed. In logistic regression model development, significant variables from the bivariate analyses (at *p* < 0.05) were included in the model. The statistics package that was used was SAS 9.3® (Cary, NC).

## 3. Results

Table 1 has the results of the sample description. There were 5758 eligible participants, 50.1% of whom were female. Considering race/ethnicity, 68.1% of participants were non-Hispanic white, 10.6% of participants were non-Hispanic black, and 8.4% of participants were Mexican American. There were 41.9% of participants who were aged 30–44 years; 28.7% of participants who were 45–54 years; and 29.4% of participants who were 55–69 years. There were 64.2% of participants who attended some college courses, technical school courses, or above. Most had an income to poverty ratio above 2.0 (69.9%). There were 56.1% of participants who were never smokers and 20.5% of participants who never used alcohol. There were 27.0% of participants who had a body mass index of 0–24, 36.0% of participants who had a body mass index of 25–29, and 37.0% of participants who had a body mass index of 30 and above. There were 12.1% of participants who had ≥40 units/liter Alanine Aminotransferase and 39.9% of participants who had periodontitis.

The Chi-square bivariate analyses and standard errors are presented in Table 2. There was a significant relationship (*p* = 0.0360) of serum Alanine Aminotransferase levels ≥40 U/L and periodontitis. Other significant relationships (at *p* < 0.0001) were with periodontitis and sex; race/ethnicity; age; education; family income to poverty ratio; alcohol consumption; smoking status; insurance coverage; and diabetes mellitus. Body mass index was also significant at *p* = 0.0151.

Table 3 has the results of the logistic regression of serum Alanine Aminotransferase on periodontitis. The odds ratio, in unadjusted logistic regression, for periodontitis associated with higher levels of serum Alanine Aminotransferase was 1.30 (95% confidence interval: 1.02, 1.65).

An Adjusted Model was created which included sex (male versus female), race/ethnicity, (non-Hispanic black, Mexican American, and other versus non-Hispanic white), education (high school, less than high school, and some college versus college degree and above), age (45–54, 55 years and above versus 30–44 years), family income to poverty ratio (less than 2 versus 2 and above), smoking status (current, former smoker versus never smoker), alcohol consumption (moderate, heavy versus no consumption), body mass index (25–29, 30 and above versus 0–24), insurance coverage (no versus yes), and diabetes (yes versus no). The adjusted odds ratio for periodontitis associated with higher levels of serum Alanine Aminotransferase was 1.17 (95% confidence interval: 0.85, 1.60).

## 4. Discussion

In this study we evaluated the relationship of Alanine Aminotransferase on periodontitis in a large, nationally representative sample of 5758 participants. The results of this study included an initial significant association of Alanine Aminotransferase with periodontitis (odds ratio 1.30, 95% CI, 1.02, 1.65), which remained positive but attenuated and failed to reach significance with the addition of other variables (adjusted odds ratio 1.17, 95% CI, 0.85, 1.60). Serum Alanine Aminotransferase was dichotomized at <40 units/liter (the reference group) and ≥40 units/liter in this study, although other researchers have used lower cut-points, such as ≥30 units/liter [24], and some researchers have used a higher level such as ≥41 units/liter [18]. When a cut-point of ≥30 units/liter was used with these data, the adjusted odds ratio was 1.16 (95% CI, 0.90, 1.50) and when a cut-point of ≥41 units/liter was used with these data, the adjusted odds ratio was 1.26 (0.89, 1.77).

The results of this study are similar to the results of researchers studying* salivary* Alanine Aminotransferase in a sample of 50 participants in Romania. They determined that salivary Alanine Aminotransferase was not statistically different among individuals who had periodontitis as compared with individuals who did not have periodontitis [25]. The study in Romania differed from this current study in the source of Alanine Aminotransferase (saliva rather than blood or serum) and study population size.

It has been suggested that changes in enzymatic activity indicate metabolic change in inflammation and that Alanine Aminotransferase release increases with cell injury and death. Researchers in a study of 49 older adult Japanese patients with chronic periodontitis found Alanine Aminotransferase to be associated with periodontal inflammation [26]. Several of the same researchers conducted an 18-month longitudinal study of 85 patients with chronic periodontitis in four Japanese university hospitals and found that* salivary* Alanine Aminotransferase in combination with the ratio of* Porphyromonas gingivalis* to total bacteria had a sensitivity of 0.4 and a specificity of 0.96 for the prediction of the progression of periodontitis [27].* Salivary* Alanine Aminotransferase was increased in individuals with periodontitis disease, as compared with controls in a study of 40 patients in India [28]. Researchers found a correlation of salivary Alanine Aminotransferase and periodontitis in a sample of 60 participants [28]. These existing studies differed from the current study in that they were small and the researchers used* salivary* Alanine Aminotransferase for analyses.

In a study conducted in Japan with* serum* Alanine Aminotransferase, a positive association of elevated serum Alanine Aminotransferase and pocket depth was present only in males with low alcohol consumption and at least 3 components of the metabolic syndrome (waists at or above 90 cm in males and 80 cm in females; triglycerides at or above 150 mg/dL or treatment for triglycerides; high density lipoprotein below 40 mg/dL in males or below 50 mg/dL for females; systolic blood press at or above 130 mmHg or diastolic blood pressure at or above 85 or medication for high blood pressure; and fasting glucose at or above 100 mg/dL or treatment for elevated glucose) [29].

Researchers who conducted a systematic review with the association of Alanine Aminotransferase on mortality indicated that Alanine Aminotransferase had a complex and inconsistent relationship, which was more predictive in older adults with extremely low levels of Alanine Aminotransferase [30]. The method of collection of the Alanine Aminotransferase was not discussed in the review of the 12 articles selected for the meta-analysis [30].

### 4.1. Strengths, Limitations, and Sources of Bias

Research concerning Alanine Aminotransferase and periodontitis is lacking. Alanine Aminotransferase collection methods differ among researchers and researchers have used different cut-points for Alanine Aminotransferase as well as different definitions for periodontitis.

Our study strengths are its large, nationally representative sample; the use of definitions for population-based surveillance of the CDC in partnership with the American Academy of Periodontology; standardized blood testing procedures for Alanine Aminotransferase; and standardized/calibrated examiners. Since this study is large, many variables were included in the multivariable analysis which are also independently related to periodontitis or may confound the relationship of Alanine Aminotransferase and periodontitis, an option that is not available for small studies.

Our study limitations are the biases associated with self-reporting of education, income, insurance status, alcohol consumption, and the reporting of diabetes mellitus. It is possible that self-reports may be influenced by participants' desires for providing a socially desirable response. However, it would be expected that social desirability bias would likely result in reports which would tend to direct the outcome to supporting the null hypothesis that the odds ratio for the association of high Alanine Aminotransferase and periodontitis is the same as the odds ratio for normal levels of Alanine Aminotransferase and periodontitis. Another limitation was not having enough participants to further refine periodontitis as “mild,” “moderate,” and “severe.”

## 5. Conclusion

Our study adds to the literature a positive but attenuated association of periodontitis and Alanine Aminotransferase which failed to reach significance when other known, strong factors of periodontitis were included in the analysis.

## Figures and Tables

**Table 1:** Sample characteristics, NHANES 2009–2012, *n* = 5758.

	Number	Weighted percentage
Alanine Aminotransferase (serum)		
<40 units/liter (normal)	5,062	87.9
≥40 units/liter	696	12.1
Periodontitis		
No	2,946	60.1
Yes	2,812	39.9
Mild	207	3.0
Moderate	2,048	30.1
Severe	557	6.9
Sex		
Male	2,884	49.9
Female	2,874	50.1
Race/ethnicity		
Non-Hispanic white	2,308	68.1
Non-Hispanic black	1,231	10.6
Mexican American	932	8.4
Other	1,287	13.0
Age		
30–44 years	2,407	41.9
45–54 years	1,536	28.7
55–69 years	1,815	29.4
Education		
Less than HS	1,393	15.5
HS graduate	1,218	20.4
Some coll./tech.	1,613	29.6
Coll./tech. graduate	1,527	34.6
Family income to poverty ratio		
0 to less than 2.00	2,417	30.1
2.00 and above	2,853	69.9
Alcohol consumption		
Nonuse	1,293	20.5
Moderate use	2,041	51.1
Heavy use	1,391	28.4
Smoking status		
Current	1,219	19.0
Former	1,307	24.9
Never	3,229	56.1
Body mass index		
0–24	1,478	27.0
25–29	1,995	36.0
30 and above	2,252	37.0
Insurance		
Yes	4,178	79.3
No	1,580	20.7
Diabetes		
Yes	732	18.0
No	2,394	82.0

SE: standard error of row percent; HS: high school; and coll./tech.: college technical school.

**Table 2:** Periodontitis status in Rao Scott Chi-square analysis, NHANES, 2009–2012.

	Frequency negative	Weighted row%	Frequency Positive	Weighted row%	SE	*p* value
Alanine Aminotransferase (serum)						0.0360
<40 units/liter (normal)	2623	60.8	2439	39.2	1.5	
≥40 units/liter	323	54.5	373	38.2	3.1	
Sex						<0.0001
Male	1185	50.9	1699	49.1	1.5	
Female	1761	69.2	1113	30.8	1.7	
Race/ethnicity						<0.0001
Non-Hispanic white	1445	65.9	863	34.1	1.9	
Non-Hispanic black	502	45.5	729	54.5	2.5	
Mexican American	350	41.4	582	58.6	1.8	
Other	649	53.1	638	46.9	2.1	
Age						<0.0001
30–44 years	1602	70.0	805	27.0	1.5	
45–54 years	715	55.7	821	44.3	2.2	
55–69 years	629	45.8	1186	54.2	2.1	
Education						<0.0001
Less than HS	446	35.7	947	64.6	1.6	
HS graduate	529	51.1	689	48.9	2.4	
Some coll./tech.	884	59.6	729	40.4	1.9	
Coll./tech. graduate	1084	76.9	443	23.1	1.5	
Family income to poverty ratio						<0.0001
0 to less than 2.00	986	45.8	1431	54.1	1.6	
2.00 and above	1753	67.1	1100	32.9	1.6	
Alcohol consumption						<0.0001
Nonuse	643	57.9	659	42.1	2.4	
Moderate use	1257	68.8	784	31.2	1.8	
Heavy use	587	50.2	804	40.8	2.3	
Smoking status						<0.0001
Current	400	35.3	819	64.7	1.9	
Former	620	58.3	687	41.7	2.5	
Never	1926	69.3	1303	30.7	1.3	
Body mass index						0.0151
0–24	805	63.2	673	36.8	1.8	
25–29	1014	60.2	981	39.8	1.6	
30 and above	1109	57.7	1143	42.3	2.0	
Insurance						<0.0001
Yes	2346	64.6	1832	35.4	1.5	
No	600	42.7	980	57.3	2.0	
Diabetes						<0.0001
Yes	244	48.0	488	59.2	3.2	
No	1299	50.7	1095	38.2	1.9	

SE: standard error of row percent; HS: high school; and coll./tech.: college or technical school.

**Table 3:** Logistic regression of serum Alanine Aminotransferase on periodontal status: NHANES, 2009–2012.

	Unadjusted OR (95% CI)	Adjusted OR (95% CI)
Periodontitis		
Yes	1.30 (1.02, 1.65)	1.17 (0.85, 1.60)
No	1.00 (reference)	1.00 (reference)
Sex		
Male		2.55 (2.07, 3.15)
Female		1.00 (reference)
Race/ethnicity		
Non-Hispanic white		1.00 (reference)
Non-Hispanic black		2.07 (1.50, 2.86)
Mexican American		2.21 (1.52, 3.23)
Other		1.63 (1.13, 2.37)
Education		
Less than high school		2.62 (1.67, 4.12)
High school		1.71 (1.20, 2.44)
Some college		1.31 (0.94, 1.82)
College degree or above		1.00 (reference)
Family income to poverty ratio		
Less than 2.00		1.36 (1.06, 1.73)
2.00 and above		1.00 (reference)
Insurance coverage		
No		1.74 (1.14, 2.68)
Yes		1.00 (reference)
Body mass index		
0 to less than 25		1.00 (reference)
25 to less than 30		0.98 (0.66, 1.46)
30 and above		0.92 (0.63, 1.32)
Smoking		
Yes		3.26 (2.28, 4.68)
No		1.00 (reference)
Alcohol consumption		
Heavy		0.92 (0.63, 1.34)
Moderate		0.66 (0.47, 0.93)
None		1.00 (reference)
Diabetes		
Yes		1.32 (0.93, 1.87)
No		1.00 (reference)

OR: odds ratio; AOR: adjusted odds ratio; CI: confidence interval.
